# Updates on Diagnostic and Therapeutic Management of Gastrointestinal and Pancreatic NET

**DOI:** 10.3390/cancers14112628

**Published:** 2022-05-26

**Authors:** Sebastian Krug, Jörg Schrader, Anja Rinke

**Affiliations:** 1 Clinic for Internal Medicine I, Martin-Luther University Halle/Wittenberg, Ernst-Grube-Strasse 40, D 06120 Halle, Germany; 2 Medical Department, University Medical Center Hamburg-Eppendorf, Martinistrasse 52, D 20246 Hamburg, Germany; jschrader@uke.de; 3 Department of Gastroenterology and Endocrinology, University Hospital Marburg, Baldinger Strasse, D 35043 Marburg, Germany; sprengea@uni-marburg.de

Gastroenteropancreatic neuroendocrine neoplasms (GEP-NEN) comprise a plethora of distinct molecular-pathological, clinical, diagnostical and therapeutical approaches to enable individualized treatment. Although the incidence is low when compared to other gastrointestinal malignancies (<10/100,000), the prognosis for local and metastatic stages is considerably better and the prevalence has, therefore, risen significantly in the last 20 years [1]. With the standardization of the classification system of GEP-NEN by the latest WHO classification of 2019, it has been achieved to better define the risk groups of patients [2]. Along with the staging based on the TNM classification according to ENETS/UICC, there are strong parameters that are obtainable to determine overall prognosis [3]. In recent years, progress has also been achieved in understanding tumor biology of NEN, especially of well-differentiated pancreatic NET (PanNET). To date, for localized PanNET, a tumor size of 2 cm or below advocated between surgical resection or even surveillance [4]. However, recently published data uncovered new promising prognostic markers. Sporadic PanNET often harbor somatic DAXX/ATRX (death domain-associated protein/alpha-thalassemia/mental retardation X-linked chromatin remodeler) and MEN-1 mutations [5]. Additionally, loss of DAXX/ATRX protein expression is significantly associated with activation of alternative lengthening telomeres (ALT), which can be reflected by using telomere-specific fluorescence in situ hybridization (FISH) [6]. In PanNET the coincidence of DAXX/ATRX loss and ALT up-regulation negatively impacts the recurrence rate after curative resection and correlates with distant metastasis [6,7]. For non-metastatic small PanNET ≤2 cm relapse occurred frequently in DAXX/ATRX loss and/or ALT positivity and the recurrence-free survival (RFS) was shorter [8]. Although this holds true only for a subset of patients, evaluation of DAXX/ATRX and ALT should be implemented in clinical routine for non-metastatic PanNET. Moreover, DAXX/ATRX and ALT are highly specific conserved markers for pancreatic origin and can be utilized to uncover the primary tumor in NET metastasis of unknown origin [8]. 

Further advances in the field of NET care include molecular imaging procedures. Besides the well-established somatostatin receptor (SSTR) imaging with ^68^Ga-DOTATOC/-TATE and –NOC, the radioisotope ^64^Cu (copper) can be linked to DOTATATE with equivalent high image quality and diagnostic accuracy. Alternative approaches include SSTR antagonist such as ^68^Ga-OPS202 also called ^68^Ga-NODAGA-JR11 [9]. Preliminary data indicate a high metastasis detection rate and superior sensitivity compared to agonists in NET patients. Here, further clinical studies are warranted to decide which approach will remain or serve as the new standard in future. In patients with insulinomas SSTR imaging often fails due to low SSTR expression. Therefore, the preoperative identification of the hormone-secreting lesion often is challenging. Investigations regarding the glucagon-like peptide-1 receptor (GLP-1R) introduced exendin-4 a GLP-1 analogue which can be coupled to ^68^Ga-DOTA [10]. The ^68^Ga-DOTA-exendin-4 PET/CT is superior to conventional CT or MRI imaging and ^68^Ga-DOTATOC in patients with suspected undetectable insulinoma and is the method of choice if available. 

From a therapeutic perspective, several novel targeted therapies are in clinical development for the treatment of neuroendocrine tumors [11]. The main concept of these tyrosine kinase inhibitors (TKI) is to interrupt signaling pathways involved in mediating cell proliferation and angiogenesis. However, since the tumor microenvironment, in particular tumor-associated macrophages (TAM) were characterized as co-player for tumor progression and modulator of resistance for anti-angiogenic therapies [12,13], novel TKI such as surufatinib and cabozantinib (CABINET study, NCT 03375320) are in the research focus [14]. By additional targeting of the colony-stimulating factor receptor 1 and TIE2 receptor both compounds simultaneously can disrupt angiogenesis and induces TAM depletion thus enhancing anti-tumor efficacy. In this context, further clinical studies will determine the impact of TKI in NEN.

The Special Issue “Updates on Diagnostic and Therapeutic Management of Neuroendocrine Neoplasms” reports in 2 reviews on current developments in the multimodal therapy of PanNET as well as on new therapeutic approaches including immunotherapy, CAR-T cell therapy and vaccines in NEN [15,16]. Other original papers address the value of salvage PRRT, cell-free DNA (cfDNA) as a biomarker in NEN, and the best surgical approach for Zollinger-Ellison syndrome (ZES) in multiple endocrine neoplasia type 1 (MEN1) patients [17,18,19]. 
